# Chronic Intracortical Recording and Electrochemical Stability of Thiol-ene/Acrylate Shape Memory Polymer Electrode Arrays

**DOI:** 10.3390/mi9100500

**Published:** 2018-09-29

**Authors:** Allison M. Stiller, Joshua Usoro, Christopher L. Frewin, Vindhya R. Danda, Melanie Ecker, Alexandra Joshi-Imre, Kate C. Musselman, Walter Voit, Romil Modi, Joseph J. Pancrazio, Bryan J. Black

**Affiliations:** 1 Department of Bioengineering, The University of Texas at Dallas, Richardson, TX 75080, USA; Joshua.usoro@utdallas.edu (J.U.); Christopher.frewin@utdallas.edu (C.L.F.); vxd160030@utdallas.edu (V.R.D.); Alexandra.Joshi-imre@utdallas.edu (A.J.-I.); kate.musselman@utdallas.edu (K.C.M.); joseph.pancrazio@utdallas.edu (J.J.P.); bjb140530@utdallas.edu (B.J.B.); 2 Qualia, Inc., Dallas, TX 75252, USA; walter.voit@utdallas.edu (W.V.); romil@qualiamedical.com (R.M.); 3 Department of Materials Science and Engineering, The University of Texas at Dallas, Richardson, TX 75080, USA; Melanie.ecker@utdallas.edu

**Keywords:** intracortical implant, microelectrodes, softening, immunohistochemistry, immune response, neural interface, shape memory polymer

## Abstract

Current intracortical probe technology is limited in clinical implementation due to the short functional lifetime of implanted devices. Devices often fail several months to years post-implantation, likely due to the chronic immune response characterized by glial scarring and neuronal dieback. It has been demonstrated that this neuroinflammatory response is influenced by the mechanical mismatch between stiff devices and the soft brain tissue, spurring interest in the use of softer polymer materials for probe encapsulation. Here, we demonstrate stable recordings and electrochemical properties obtained from fully encapsulated shape memory polymer (SMP) intracortical electrodes implanted in the rat motor cortex for 13 weeks. SMPs are a class of material that exhibit modulus changes when exposed to specific conditions. The formulation used in these devices softens by an order of magnitude after implantation compared to its dry, room-temperature modulus of ~2 GPa.

## 1. Introduction

Successful clinical application of brain‒machine interfaces (BMIs) requires stable, chronic, selective recordings from task-associated neural networks. Noninvasive techniques for the acquisition of neural signals include electroencephalography and electrocorticography [1,2,3]; however, these methods cannot achieve high-density, single-unit resolution, and are therefore limited as high information content BMI systems [4,5,6]. Multichannel intracortical microelectrode arrays (MEAs) are able to record single units and local field potentials from adjacent neural tissue within the brain. While several styles of intracortical MEAs are commercially available, they are limited in clinical implementation due to a relatively short functional lifetime, only recording distinguishable units in non-human primates for an average of 1‒6 years post-implantation [7,8,9,10].

While there are various factors that contribute to MEA failure, findings suggest that the tissue response may be one component that contributes to the premature loss of stable neural recordings [11,12,13,14]. The chronic foreign body response stems from the recruitment of activated support cells to the injury site, initiating signaling cascades that result in upregulated local production of inflammatory and neurotoxic cytokines [14,15]. This leads to the accumulation of glial cells around the implant (i.e., encapsulation) concurrent with local neuronal death [11,15,16,17,18], both of which are obstacles for reliable signal acquisition.

State-of-the-art, commercially available devices are fabricated using very stiff (high elastic modulus) materials, such as silicon or tungsten (50–400 GPa). Current research suggests that this mechanical mismatch between the low modulus of the brain (~1–10 kPa) and the high modulus of the device may play a major role in aggravating the chronic immune response [17,19,20]. This effect is exacerbated by constant micromotion of the brain around the implant [21], which results in the development of strain fields in tissue adjacent to the probe [22,23]. Several groups have demonstrated that softer implants may mitigate the tissue response over time when compared to stiffer counterparts [24,25]. However, these materials are often too soft to provide appropriate mechanical support for successful penetration into the brain tissue without the aid of insertion guides [26,27]. Conversely, stiffer devices are brittle and prone to fracture, making them difficult to handle in a clinical setting.

Shape memory polymers (SMPs) are a class of materials that undergo dramatic programmed mechanical deformations or modulus changes when exposed to external stimuli such as light, electric currents, or heat [28,29,30]. Recently, softening thiol-ene/acrylate-based SMPs, which exhibit changes in modulus when transitioning from ambient to physiological conditions, have been investigated for their use as substrate and encapsulation materials for neural interfaces [31,32]. Specifically, this type of SMP can maintain a high modulus and mechanical stability necessary for the implantation of a thin device, but softens by an order of magnitude within only a few minutes [33]. However, to date, no published study has evaluated the chronic recording and electrochemical performance of fully encapsulated thiol-ene/acrylate-based SMP MEAs in vivo. To address this issue, we have implanted 15-channel Michigan-style SMP devices in the motor cortex of five rats and conducted electrophysiological recordings as well as electrochemical impedance spectroscopy (EIS) and cyclic voltammetry (CV) over a 13-week period. Additionally, we have performed immunohistochemistry (IHC) to evaluate tissue response. Our results demonstrate that these SMP devices consistently recorded units for 13 weeks and induced a minimal immune response in the surrounding tissue.

## 2. Materials and Methods

### 2.1. Shape Memory Polymer Devices

**Figure 1:**
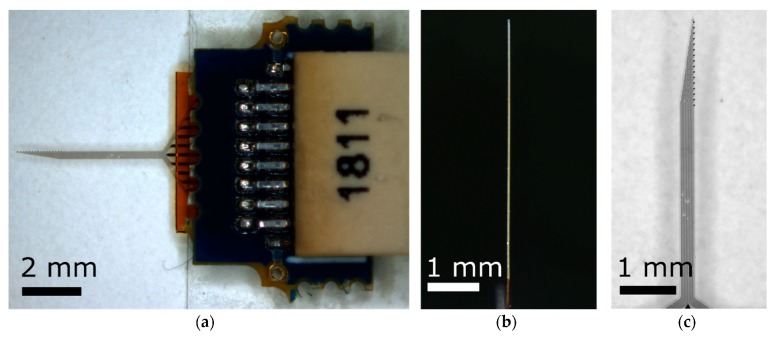
Optical images of shape memory polymer (SMP) probes. (**a**) Device with Omnetics connector, (**b**) side view demonstrating a straight shank prior to implantation, (**c**) tip with sputtered iridium oxide film (SIROF)-coated electrodes.

All experiments were carried out using IC-5-16E devices (Figure 1) provided by Qualia, Inc. (Dallas, TX, USA). Devices featured 15 electrode sites coated with sputtered iridium oxide film (SIROF) with an electrode area of 180 µm^2^. Parylene C encapsulated the thin metal traces to ensure proper electrical insulation. Shanks were 5 mm long, 290 µm wide at the base, and 35 ± 5 µm thick, with an asymmetric geometry that tapered toward the device tip. The SMP formulation used in these devices softens by an order of magnitude from its dry room-temperature modulus of ~2 GPa to ~300 MPa after implantation. This transition occurs within a few minutes of implantation.

### 2.2. Surgical Implantation

All animal handling, housing, and surgical procedures were approved by the University of Texas Institutional Animal Care and Use Committee. Long Evans rats (*n* = 5, Charles River), weighing 300–450 g, were implanted with functional SMP devices. Devices underwent brief electrochemical impedance testing before implantation to ensure all electrode sites were below 1 MΩ at 1 kHz frequency. Animals were anesthetized by an intraperitoneal (IP) injection of KXA cocktail consisting of ketamine (65 mg/kg), xylazine (13.33 mg/kg) and acepromazine (1.5 mg/kg) followed by an intramuscular injection of atropine sulfate (0.05 mg/kg) to counteract the cardiovascular depression induced by KXA. After reaching a deep anesthesia plane, confirmed by tail and toe pinches, the scalp was shaved using small hair clippers. Ophthalmic ointment was applied to the animal’s eyes to mitigate drying and post-operative irritation. The anesthetic plane was supplemented and maintained using 1–2% isoflurane mixed with 100% oxygen for the duration of the surgery.

Three alternating rounds of 10% iodine solution and 70% ethanol, ending with ethanol, were used to sterilize and clean the point of incision on the scalp. Dexamethasone was then administered subcutaneously between the shoulders (2 mg/kg), followed by subcutaneous injection of 0.4 mL 0.5% lidocaine at the incision cite. A surgical blade was used to make a midline incision down the scalp and the surrounding skin and muscle were retracted with hemostatic forceps. All loose tissue and debris was removed from the skull surface using sterile cotton swabs, and the skull was roughened using the surgical blade to promote binding of the head cap post-surgery.

A surgical drill was used to create a 1–2 mm^2^ craniotomy centered in the right motor cortex, approximately 2.5 mm rostral and 2.5 mm lateral from bregma. Three anchoring screws were positioned approximately 1 cm from the perimeter of the insertion site. The dura was resected and the device was implanted at 1000 μm/s to a depth of 1.5–2 mm using a pneumatically controlled micropositioner (Kopf Instruments, Tujunga, CA, USA). No significant curvature or bending of the device was observed prior to or during implantation. Collagen-based dural grafts (Biodesign Dural Graft, Cook Medical, Bloomington, IN, USA) were placed around the implanted device to act as a dura replacement, and then set in place with Gluture topical adhesive (World Precision Instruments, Sarasota, FL, USA). We applied dental cement around the device and all three anchoring screws to construct a protective head cap, promoting chronic mechanical stability. Before being removed from the isoflurane, the animal was given 0.15 mg/kg of sustained release buprenorphine SR LAB (ZooPharm, Windsor, CO, USA) and 5 mg/kg of cefazolin antibiotic along with subcutaneous sterile saline to prevent dehydration. All animals received follow-up analgesic injections of buprenorphine SR 72 h following surgery. None of the animals showed signs of post-operative complications including chronic bleeding, signs of infection, or skin ulcers.

### 2.3. Electrophysiological Recordings and Single-Unit Analysis

Electrophysiological recordings were carried out on lightly anesthetized animals (0.5–1.5% isoflurane) immediately following surgical implantation and once per week for 13 weeks afterward. Spontaneous wideband recordings (0.1–7000 Hz) were collected using 15-channel Michigan style SMP arrays (IC-5-16E, Qualia, Inc.) and an Omniplex acquisition system (Plexon, Inc., Dallas, TX, USA) from all 15 recording sites simultaneously at 40,000 Hz for 10 min. Wideband data were processed using a four-pole Butterworth high pass filter with a cutoff frequency of 250 Hz. Individual waveforms (spikes) were identified by filtered continuous data crossing a threshold of −4σ, based on the root mean square (RMS) of the filtered continuous signal. Single units were manually identified from collections of spikes using 2D principal component space, but were excluded from further analysis if they did not contain at least 100 individual spikes, or if >3% of spikes violated a 1.5-ms minimum refractory period. The signal to noise ratio (SNR) was calculated by dividing the mean peak-to-peak voltage of each unit (Vpp) by the RMS noise of its associated channel. The RMS noise was calculated as the RMS of the filtered continuous signal after removing all samples exceeding the 4σ threshold.

### 2.4. EIS and CV Measurements

EIS and CV measurements were carried out on all electrodes each week immediately following in vivo electrophysiological recordings. The Plexon headstage was removed and replaced with a pre-wired 18 pin dual strip Nano-D female connector (NSD-18-WD-18.0-C-GS, Omnetics Connector Corporation, Minneapolis, MN, USA) attached to multiplexor inputs of a model 604E Series Electrochemical Analyzer/Workstation (CH Instruments Inc., Austin, TX, USA). EIS was performed using a 10 mV RMS sinusoidal signal (Vrms), starting at a frequency of 100 kHz and decreasing to 1 Hz, recording current 12 times per decade of frequency. The impedance magnitude at each frequency was calculated by the CH instruments software. CV evaluations were performed by applying a negative potential ramp starting at the open circuit potential (vs. 316 stainless steel) with no external direct current bias applied. The potential was reduced to −0.6 V and then cycled at 50 mV/s for two complete cycles between −0.6 V and 0.8 V while recording current every 10 ms. A second CV measurement was performed under the same conditions, but at a sweep rate of 50,000 mV/s.

Electrochemistry results were process in MATLAB to extract the values of real impedance (Ω) directly from the EIS recordings at the physiological frequencies of 0.01, 1, and 10 kHz. MATLAB scripts also determined cathodal charge storage capacity (CSCc), a measure of the total cathodal charge available per unit of geometric area, from the CV cathodal current between the limits of −0.6 to 0.8 V.

### 2.5. Immunohistochemistry

#### 2.5.1. Tissue Preparation

Rats were administered a 200 mg/kg IP injection of sodium pentobarbital. After confirming unconsciousness through tail and toe pinches, the rats were transcardially perfused with room-temperature phosphate buffered saline (PBS) followed by room-temperature 4% paraformaldehyde (PFA) solution. The brain was removed such that the device was kept intact with the connector and surrounding skull. The brain was stored in PFA at 4 °C overnight, then transferred to PBS with sodium azide and stored at 4 °C until sectioning.

Prior to sectioning, brains were submerged in a 4% (m/V) agarose solution for stability. Vibratome sections (Leica VT 1000 S, Leica Biosystems Inc., Buffalo Grove, IL, USA) were collected from the surface of the brain to a 2 mm depth (200 µm slices) and then stored in PBS with 0.1% (*w*/*v*) sodium azide (Alfa Aesar, Tewksbury, MA, USA) at 4 °C until staining.

#### 2.5.2. Antibody Staining

Brain slices were blocked in 4% (*v*/*v*) normal goat serum (Abcam Inc., Cambridge, UK) with 0.3% (*v*/*v*) Triton X-100 (Sigma-Aldrich, Saint Louis, MO, USA) in 1× PBS with 0.1% sodium azide (Alfa Aesar) for one hour. Slices were then incubated overnight with primary antibodies targeting neuronal nuclei (NeuN), astrocytes (GFAP), and activated microglia/macrophages (CD68) (Table 1) at 4 °C in a buffer solution containing only 0.1% (*v*/*v*) Triton X-100.

The following day, slices were washed and incubated for one hour in blocking solution with secondary antibodies, goat anti-rabbit IgG (TRITC), goat anti-mouse IgG (Alexa Fluor 488), goat anti-chicken IgY (Alexa Fluor 647), at 1:1000 dilution, and DAPI (0.6 μM) (Abcam Inc.). Slices were subsequently washed and mounted on glass slides with Fluoromount aqueous mounting medium (Sigma-Aldrich).

#### 2.5.3. IHC Imaging

Stained tissue slices were imaged using an inverted confocal microscope (Nikon Ti eclipse + A1R, Tokyo, Japan) controlled by Nikon Instruments Software package (version AR 4.40.00). Briefly, z-stack images were collected at 1024 × 1024 transverse resolution and 5 µm per axial slice using a 20× Ph2 objective. Fluorescence signal-to-noise was increased by enabling 2× pixel averaging and bleed-over between emission lines was reduced by collecting each emission line in series. All microscope hardware and software settings were conserved between individual image collections and imaging sessions. Following acquisition, z-stack images were collapsed to single maximum intensity projection image.

#### 2.5.4. IHC Quantification

Astrogliosis (GFAP intensity) and neuronal density were quantified as described in [34]. Briefly, images were imported into Fiji [35], and open source imaging software based on ImageJ [36]. Using a custom macro, GFAP intensities and NeuN+ nuclei per area were calculated within at least eight concentric bands of 50 µm thickness generated from a user-defined implant site. All reported values were normalized to measurements from the band located 350–400 µm from the device edge.

### 2.6. Statistical Analysis

Statistical analysis and graphing were carried out in OriginPro 2017 (Origin Lab, Northampton, MA, USA). In all cases, statistical significance of increasing/decreasing differences (*p* < 0.05) was determined by carrying out analysis of variance (ANOVA) tests on residuals. In the case of both EIS and CV measurements, a single-tiered Grubb’s test was applied at a 0.05 significance level to exclude aberrant statistical outliers.

## 3. Results

### 3.1. Chronic Single Unit Recordings

**Figure 2:**
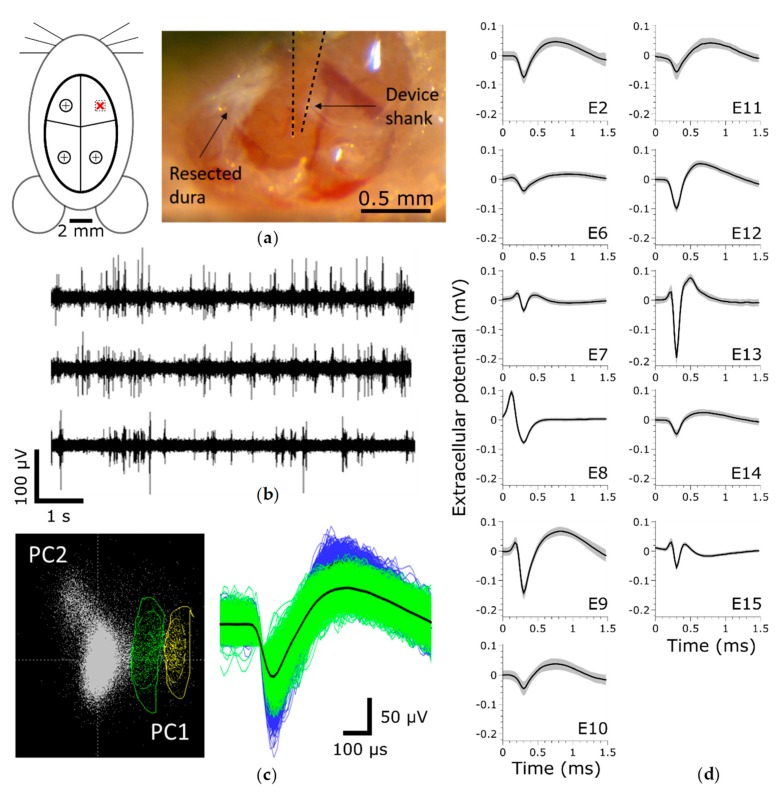
Neural data acquisition and waveform analysis. (**a**) Implantation schematic denoting the implantation site (red “x”) and stabilizing screws, (**b**) filtered continuous data from three representative electrodes on a single array, (**c**) representation of single-unit sorting principals (left) and representative multi-unit activity from a single recording electrode (right), (**d**) single units recorded on a single array during a single recording session, ordered from array tip (E2) to base (E15).

**Figure 3:**
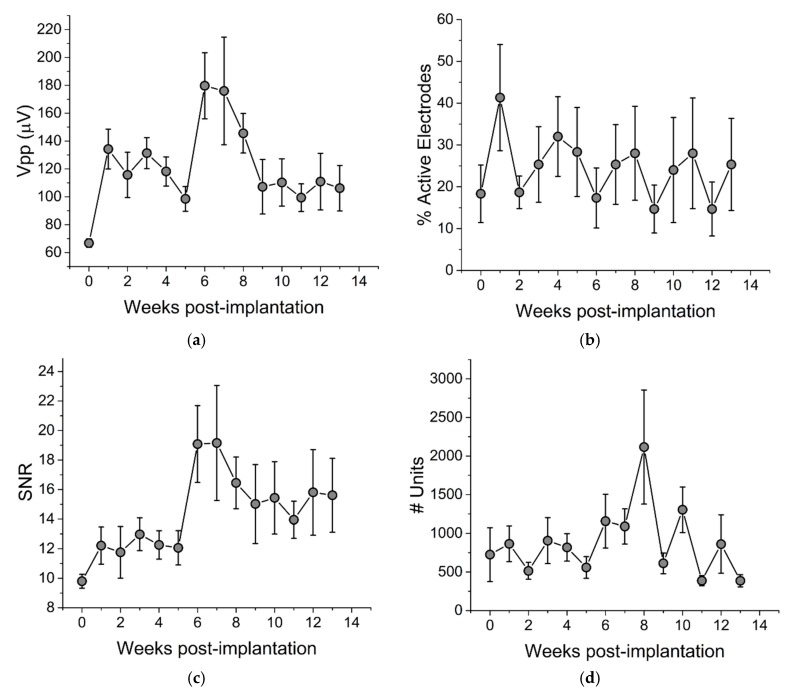
Chronic recording stability. (**a**) peak-to-peak voltage (Vpp), (**b**) active electrode yield %, (**c**) signal-to-noise ratio (SNR), and (**d**) number of units. Each time point reflects *n* = 75 electrodes, except weeks 0 and 5, which reflect *n* = 60 electrodes. Linear regression analysis indicates no change in Vpp, active electrode yield %, or SNR, while number of units increased slightly (*R*^2^ = 0.016, *p* = 0.02). Week 0 represents data taken on the day of implantation. Data are shown as mean ± SEM.

To evaluate the chronic recording performance of thiol-ene/acrylate-based SMP devices, we implanted 15-channel Michigan-style single shank electrode arrays in the motor cortex of five Long Evans rats and collected spontaneous wide-band 10-min recordings for 13 weeks post-implantation (Figure 2). Immediately following implantation, we observed that 18.3 ± 6.9% (mean ± SEM, *n* = 60) of electrode sites across all devices exhibited distinguishable single units (termed “Active electrode yield %”) (Figure 3b). At this time, the mean peak-to-peak voltage of sorted waveforms (Figure 3a) was 66.8 ± 3.1 µV, *n* = 75, resulting in an excellent mean signal-to-noise ratio (SNR) of 9.80 ± 0.47 (Figure 3c). One week post-implantation, the active electrode yield increased to 41.3 ± 12.7%. While, there was no significant change in active electrode yield or SNR over the remaining 13-week period, the total number of recorded units (Figure 2d) increased slightly (*R*^2^ = 0.02, *p* = 0.02). Overall, these data suggest that our SMP electrodes were stable with regard to their recording capabilities.

### 3.2. Chronic In Vivo Electrochemistry

**Figure 4:**
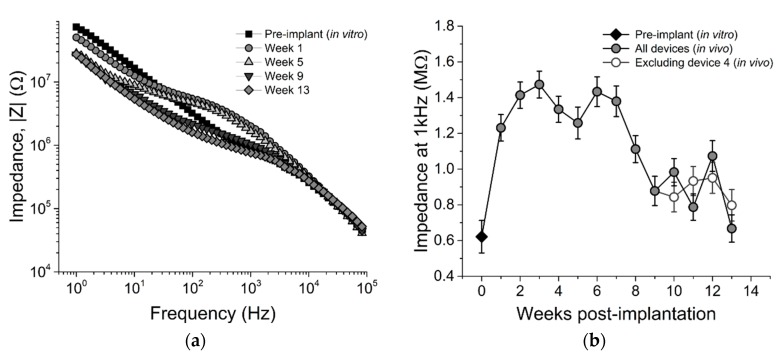
Electrochemical impedance spectroscopy. (**a**) Impedance across 1–10 kHz frequency range for a representative electrode at five time points and (**b**) impedance magnitude at 1 kHz across all electrodes on all devices. Impedance was initially low (~600 kΩ) upon testing before implantation, but increased following implantation. From week 1 until week 13, impedance magnitude decreased over time. Pre-implantation data are from *n* = 45 electrodes. All other time point reflect *n* = 75 electrodes, except weeks 0 and 5, which reflect *n* = 60 electrodes.

To evaluate the electrochemical stability of the SMP devices over time, we performed EIS and CV measurements on each array across all electrode sites for 13 weeks post-implantation. EIS and CV data are also presented for week 0 data points indicating pre-implantation measurements taken in room temperature PBS. Figure 4a shows representative traces for mean EIS for a single device prior to implantation (in vitro), immediately following implantation, and at five, nine, and 13 weeks following implantation. To evaluate the stability over a frequency relevant to extracellular spikes, the mean 1 kHz impedance across all devices is plotted in Figure 4b over the 13-week time period.

All electrodes exhibited a significant increase in impedance magnitude one week post-implantation (1.23 ± 0.07 MΩ) versus in vitro measurements (0.62 ± 0.09 MΩ), similar to observations made in [37]. Impedance magnitudes remained largely consistent at this value across the first seven weeks of the study, and then decreased slightly during the remaining six weeks. This decrease did not show any correlation with the mean active electrode yield, however, suggesting that although there may have been degradation of the insulating material, this degradation did not hinder the devices’ ability to resolve and record single unit activity.

**Figure 5:**
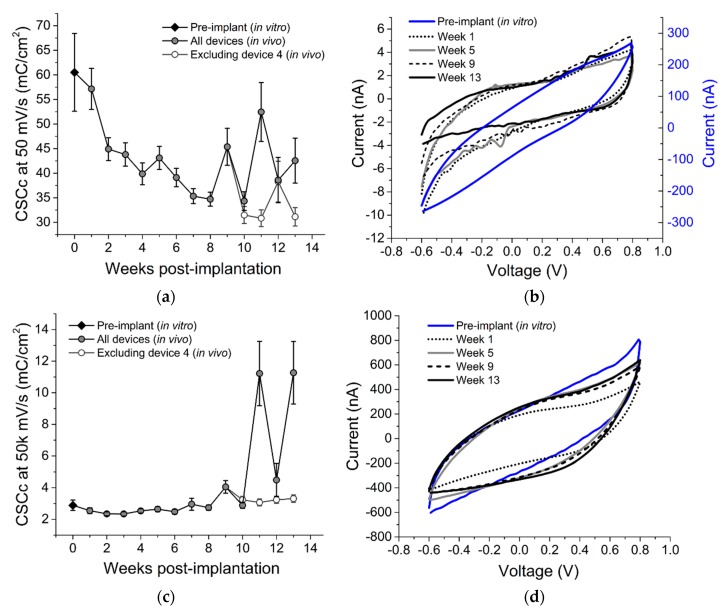
Cyclic voltammetry (CV) results. (**a**,**c**) slow (50 mV/s) and fast (50 k mV/s) CV over 13 weeks, (**b**,**d**) slow and fast CV curves across five time points on a representative electrode.

Figure 5a–d shows representative CV traces for a single electrode at two different sweep rates (50 and 50,000 mV/s) at selected time points, as well as mean CSC_C_ for both sweep rates across all devices and time points. Faster sweep rates are indicative of conductive pathways that are near the tip of the device, while slower sweep rates allow access to conductive pathways proximal to the tip.

We observed an increase over time in CSC_C_ at 50,000 mV/s, (*R*^2^ = 0.07, *p* < 0.001) likely indicating increased access to conductive paths that could be due to cracks or separation between the insulating and conductive layers near the tip. CSC_C_ at 50 mV/s decreased within the first two weeks post-implantation as compared to in vitro measurements, and continued to decrease until week 8, suggesting a possible loss of the SIROF coating. After week 10, however, the CSC_C_ increased. This change was attributable to values recorded from a single device (device 4), as demonstrated in Figure 5a,c, and also supported by EIS results (Figure 4b). Nevertheless, the majority of the devices exhibited stable electrochemistry over time, as also supported by the stable neural recordings.

### 3.3. Histology

**Figure 6:**
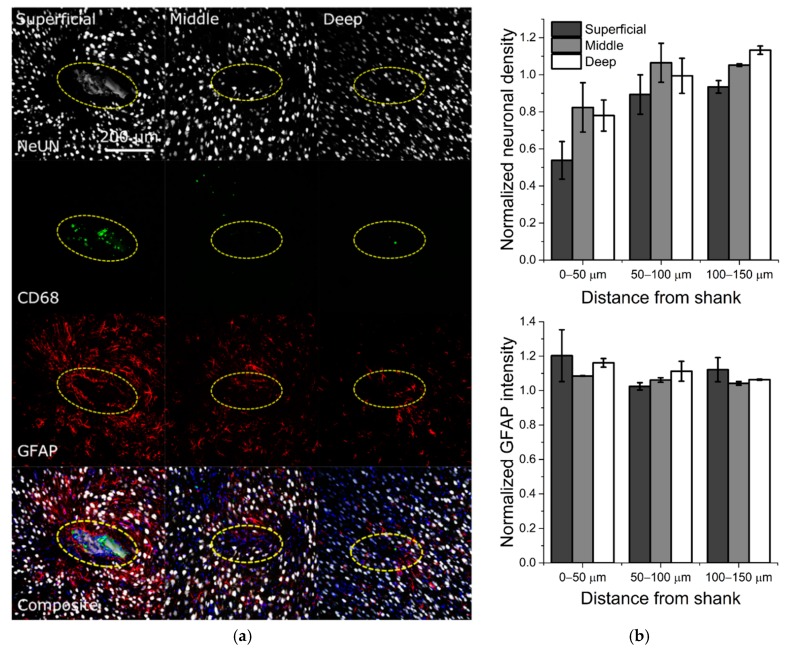
Immunohistochemistry for SMP implant after 13 weeks in vivo. (**a**) Columns represent tissue collected at superficial, middle, and deep slices in relation to the surface of the motor cortex. Rows represent neuronal nuclei (NeUN) (gray, top), activated microglia/macrophages (CD68) (green), astrocytes (GFAP) (red), and Composite (bottom) images. Scale bar represents 200 µm in the transverse plane across all images. Yellow ellipses indicate probe location. (**b**) Quantification of NeuN and GFAP for *n* = 2 animals at varying slice depths (one slice per region) with respect to the shank location in the brain.

To evaluate the induced FBR related to chronic implantation of softening SMP devices, we performed histology targeting neuronal cell bodies (NeuN), astrocytes (GFAP), and activated microglia/macrophages (CD68). Figure 6a shows representative fluorescence images for each marker with respect to increasing depth along the shank of the device.

While Figure 6 demonstrates promising histological outcomes, these results are preliminary and require a more comprehensive analysis comparing SMP with standard silicon devices to make statistical claims. The most severe apparent immune response was observed at the base of the shank, represented by “superficial” slices, within the first 50 µm of the device perimeter (Figure 6b). However, this effect tapered off along the length of the probe, represented by “middle” and “deep” slices. Additionally, consistent with previous studies reporting histological outcomes, we observed slight astrogliosis in areas with neuronal dieback, again with the most severe response near the base of the shank but tapered off toward the tip. This is in contrast to previous reports of significant neuroinflammatory response within 100 µm of the device when using silicon-based arrays [15]. Additionally, there were few or no apparent activated microglia around the device at “middle” and “deep” slices. It is important to note that the microelectrode sites on this device are located near the end of the device shank, and therefore best represented by the “middle” and “deep” slices. Therefore, it appears that the neuroinflammatory response was modest proximal to the microelectrode site locations.

## 4. Discussion

Significant efforts have been directed toward developing penetrating intracortical MEAs that mitigate the FBR. Prior work has made use of three general strategies or combinations thereof: (1) decreasing MEAs dimensions [38,39], (2) utilizing soft or softening materials for MEAs [24,25], and (3) coating MEAs with biomimetic gels, proteins, or growth factors to depress the foreign body response or facilitate local regeneration [40,41]. While these approaches have all yielded varying levels of success in terms of histological response, the chronic recording reliability of penetrating intracortical MEAs remains a significant challenge. Here, for the first time, we have demonstrated stable chronic recordings from a fully encapsulated softening SMP-based electrode array implanted in the motor cortex of rats. Importantly, we observed no significant decrease in active electrode yield over a 13-week indwelling period. Additionally, we performed electrochemical measurements to evaluate the electrical stability of these arrays. While we initially observed an increase in 1 kHz impedance magnitude, most likely associated with acute inflammation, the impedance approached its pre-implantation values over time, suggesting a resolution of the acute immune response over the first eight weeks in vivo [42]. This is further supported by the limited FBR we observed following device explantation. Cathodal charge storage capacity was also found to be largely consistent over the indwelling period. However, in the case of one electrode array, the CSC increased dramatically over the final three weeks of implantation (approximately 2 orders of magnitude). Concurrently, this electrode array exhibited reduced impedances. This was most likely due to trace or wire bundle breakage.

Table 2 summarizes previous studies using state-of-the-art single-shank silicon- and SMP-based electrode arrays. While there are important differences in all these studies in terms of N number, study duration, implantation site, single-unit sorting criteria, and electrode material/deposition, our results (top row) compare well in terms of terminal AEY% with the current state of the art (25 ± 11% versus 10–59%). Additionally, there have been significant prior efforts to leverage soft or softening polymers as either an insulator or a structural material for intracortical MEAs. Luan et al. demonstrated chronic recording capability with a comparable active electrode yield (20–25%), and also exhibited minimal tissue response [43]. However, due to their extreme flexibility, they required an insertion guide, which may not be practical for applications using multi-shank structures. This highlights one of the inherent advantages of softening over soft material approaches. Other groups have investigated Parylene C [44,45] and polyimide [46], but have not achieved chronic recordings up to or longer than one month. Others have investigated softening polymers [24,25], but face significant challenges in fabricating functional devices due to water absorption during softening.

**Figure 7:**
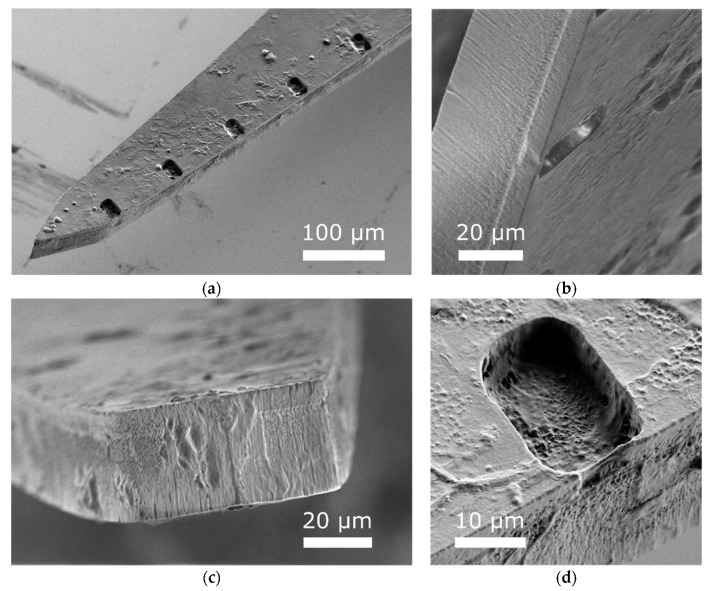
SEM post-explantation. (**a**) Explanted array showing some, but limited, biofouling, (**b**) interface between SMP layers at an electrode site, indicating no apparent layer separation, (**c**) interface between SMP layers at the tip of the array, (**d**) representative electrode site, showing no apparent signs of SIROF delamination.

The feasibility of SMP-based recording MEAs has been demonstrated in both rat auditory [31] and motor cortex [32]. The work presented here takes full advantage of SMP as an encapsulation material. Whereas previous studies have assessed devices that are SMP on one side and Parylene C on the other, the devices used here are completely sandwiched between layers of both Parylene C and SMP. In this way, we show that SMP is viable for use as a substrate material for neural device encapsulation, along with a thin layer of Parylene C necessary for electrical isolation. Additionally, SEM images collected post-explantation (Figure 7) reveal no evident signs of encapsulation failure or cracking.

Future studies focused on the design and development of SMP-based MEAs should take advantage of both geometrical and chemical considerations. For example, recently developed SMP formulations may extend the dynamic softening range of SMP devices [48]. Additionally, one inherent disadvantage of thiol-ene/acrylate polymers based on ester linkages is that they may exhibit degradation due to hydrolysis. The development of hydrolytically stable SMP formulations may provide a more chronically useful substrate. Nevertheless, for experiments over the time course of 13 weeks in vivo, the present SMP formulation appears sufficient to realize functional devices.

## 5. Conclusions

Here we demonstrated stable neural recordings and electrochemistry using IC-5-16E devices fully encapsulated with SMP. Devices consistently recorded single units for 13 weeks in the rat motor cortex and preliminary histology demonstrated only a modest tissue response in the tissue adjacent to the insertion site. Our results establish a valuable baseline for the evaluation of other softening probe technologies including devices comprised of SMPs capable of increased softening after implantation.

## Figures and Tables

**Table 1:** Primary antibodies.

Primary	Vendor	ID#	Dilution	Labeling
NeuN	Sigma-Aldrich	ABN91	1:500	Neuronal nuclei
GFAP	Millipore-Sigma	AB5541	1:500	Astrocytes
CD68	Fisher Scientific	MS397P0	1:1000	Activated microglia/macrophages

**Table 2:** Planar single-shank electrode array comparison. Active electrode yield (AEY) percentage represents spontaneous single-unit activity unless otherwise indicated.

Ref	Model (R/M), Implant Site	*N*, Study Duration	AEY%	Substrate, Electrode Material
-	R, MC	*N* = 5, 13 weeks	25 ± 11%	SMP, SIROF
[32]	R, MC	*N* = 2, 11 weeks	* 37 ± 13%	SMP, PEDOT:PSS
[47]	R, MC	*N* = 8, 6 weeks	59% (Ir, SNR > 2)	Si, Ir or PEDOT
[37]	R, MC	*N* = 5, 12 weeks	33 and 39%	Si, Au or PEDOT:TFB
[7]	R, MC	*N* = 4, 4 weeks	* 27%	Si, Ir
[48]	M, VC	*N* = 4, 27 weeks	* 10% (spontaneous)	Si, Ir

* Represents approximate or recalculated values in the case that AEY was not reported. (R/M)—Rat or Mouse model. MC—motor cortex, VC—visual cortex.

## References

[1] Kübler A., Kotchoubey B., Kaiser J., Wolpaw J.R., Birbaumer N. (2001). Brain-computer communication: Unlocking the locked in. Psychol. Bull..

[2] Obermaier B., Müller G.R., Pfurtscheller G. (2003). “Virtual keyboard” controlled by spontaneous EEG activity. IEEE Trans. Neural Syst. Rehabil. Eng..

[3] Pailla T., Jiang W., Dichter B., Chang E.F., Gilja V. ECoG data analyses to inform closed-loop BCI experiments for speech-based prosthetic applications. Proceedings of the 38th Annual International Conference of the IEEE Engineering in Medicine and Biology Society (EMBC).

[4] Birbaumer N. (2006). Brain-computer-interface research: Coming of age. Clin. Neurophysiol..

[5] Lebedev M.A., Nicolelis M.A. (2006). Brain-machine interfaces: Past, present and future. Trends Neurosci..

[6] Kipke D.R., Shain W., Buzsaki G., Fetz E., Henderson J.M., Hetke J.F., Schalk G. (2008). Advanced neurotechnologies for chronic neural interfaces: New horizons and clinical opportunities. J. Neurosci..

[7] Ward M.P., Rajdev P., Ellison C., Irazoqui P.P. (2009). Toward a comparison of microelectrodes for acute and chronic recordings. Brain Res..

[8] Barrese J.C., Rao N., Paroo K., Triebwasser C., Vargas-Irwin C., Franquemont L., Donoghue J.P. (2013). Failure mode analysis of silicon-based intracortical microelectrode arrays in non-human primates. J. Neural Eng..

[9] Barrese J.C., Aceros J., Donoghue J.P. (2016). Scanning electron microscopy of chronically implanted intracortical microelectrode arrays in non-human primates. J. Neural Eng..

[10] Suner S., Fellows M.R., Vargas-Irwin C., Nakata G.K., Donoghue J.P. (2005). Reliability of signals from a chronically implanted, silicon-based electrode array in non-human primate primary motor cortex. IEEE Trans. Neural Syst. Rehabil. Eng..

[11] Polikov V.S., Tresco P.A., Reichert W.M. (2005). Response of brain tissue to chronically implanted neural electrodes. J. Neurosci. Methods.

[12] Seymour J.P., Kipke D.R. (2007). Neural probe design for reduced tissue encapsulation in CNS. Biomaterials.

[13] Leach J., Achyuta A.K.H., Murthy S.K. (2010). Bridging the divide between neuroprosthetic design, tissue engineering and neurobiology. Front. Neuroeng..

[14] Karumbaiah L., Saxena T., Carlson D., Patil K., Patkar R., Gaupp E.A., Betancur M., Stanley G.B., Carin L., Bellamkonda R.V. (2013). Relationship between intracortical electrode design and chronic recording function. Biomaterials.

[15] Biran R., Martin D.C., Tresco P.A. (2005). Neuronal cell loss accompanies the brain tissue response to chronically implanted silicon microelectrode arrays. Exp. Neurol..

[16] McConnell G.C., Rees H.D., Levey A.I., Gutekunst C.-A., Gross R.E., Bellamkonda R.V. (2009). Implanted neural electrodes cause chronic, local inflammation that is correlated with local neurodegeneration. J. Neural Eng..

[17] Moshayedi P., Ng G., Kwok J.C., Yeo G.S., Bryant C.E., Fawcett J.W., Franze K., Guck J. (2014). The relationship between glial cell mechanosensitivity and foreign body reactions in the central nervous system. Biomaterials.

[18] Nolta N.F., Christensen M.B., Crane P.D., Skousen J.L., Tresco P.A. (2015). BBB leakage, astrogliosis, and tissue loss correlate with silicon microelectrode array recording performance. Biomaterials.

[19] Andrei A., Welkenhuysen M., Nuttin B., Eberle W. (2011). A response surface model predicting the in vivo insertion behavior of micromachined neural implants. J. Neural Eng..

[20] Karumbaiah L., Norman S.E., Rajan N.B., Anand S., Saxena T., Betancur M., Patkar R., Bellamkonda R.V. (2012). The upregulation of specific interleukin (IL) receptor antagonists and paradoxical enhancement of neuronal apoptosis due to electrode induced strain and brain micromotion. Biomaterials.

[21] Gilletti A., Muthuswamy J. (2006). Brain micromotion around implants in the rodent somatosensory cortex. J. Neural Eng..

[22] Sridharan A., Nguyen J.K., Capadona J.R., Muthuswamy J. (2015). Compliant intracortical implants reduce strains and strain rates in brain tissue in vivo. J. Neural Eng..

[23] Subbaroyan J., Martin D.C., Kipke D.R. (2005). A finite-element model of the mechanical effects of implantable microelectrodes in the cerebral cortex. J. Neural Eng..

[24] Harris J.P., Capadona J.R., Miller R.H., Healy B.C., Shanmuganathan K., Rowan S.J., Weder C., Tyler D.J. (2011). Mechanically adaptive intracortical implants improve the proximity of neuronal cell bodies. J. Neural Eng..

[25] Lee H.C., Ejserholm F., Gaire J., Currlin S., Schouenborg J., Wallman L., Bengtsson M., Park K., Otto K.J. (2017). Histological evaluation of flexible neural implants; Flexibility limit for reducing the tissue response?. J. Neural Eng..

[26] Shoffstall A.J., Srinivasan S., Willis M., Stiller A.M., Ecker M., Voit W.E., Pancrazio J.J., Capadona J.R. (2018). A mosquito inspired strategy to implant microprobes into the brain. Sci. Rep..

[27] Lo M.C., Wang S., Singh S., Damodaran V.B., Kaplan H.M., Kohn J., Shreiber D.I., Zahn J.D. (2015). Coating flexible probes with an ultra fast degrading polymer to aid in tissue insertion. Biomed. Microdevices.

[28] Mather P.T., Luo X., Rousseau I.A. (2009). Shape memory polymer research. Annu. Rev. Mater. Res..

[29] Wang K., Strandman S., Zhu X.X. (2017). A mini review: Shape memory polymers for biomedical applications. Front. Chem. Sci. Eng..

[30] Leng J., Lan X., Liu Y., Du S. (2011). Shape-memory polymers and their composites: Stimulus methods and applications. Prog. Mater. Sci..

[31] Ware T., Simon D., Liu C., Musa T., Vasudevan S., Sloan A., Keefer E.W., Ii R.L.R., Voit W. (2013). Thiol-ene/acrylate substrates for softening intracortical electrodes. Appl. Biomater..

[32] Simon D.M., Charkhkar H., St. John C., Rajendran S., Kang T., Reit R., Arreaga-Salas D., McHail D.G., Knaack G.L., Sloan A. (2017). Design and demonstration of an intracortical probe technology with tunable modulus. J. Biomed. Mater. Res. Part A.

[33] Do D.-H., Ecker M., Voit W.E. (2017). Characterization of a thiol-ene/acrylate-based polymer for neuroprosthetic implants. ACS Omega.

[34] Stiller A., Black B., Kung C., Ashok A., Cogan S., Varner V., Pancrazio J. (2018). A meta-analysis of intracortical device stiffness and its correlation with histological outcomes. Micromachines.

[35] Schindelin J., Arganda-Carreras I., Frise E., Kaynig V., Longair M., Pietzsch T., Preibisch S., Rueden C., Saalfeld S., Schmid B. (2012). Fiji: An open-source platform for biological-image analysis. Nat. Methods.

[36] Schneider C.A., Rasband W.S., Eliceiri K.W. (2012). NIH Image to ImageJ: 25 years of image analysis. Nat. Methods.

[37] Charkhkar H., Knaack G.L., Mchail D.G., Mandal H.S., Peixoto N., Rubinson J.F., Dumas T.C., Pancrazio J.J. (2016). Chronic intracortical neural recordings using microelectrode arrays coated with PEDOT-TFB. Acta Biomater..

[38] Pancrazio J.J., Deku F., Ghazavi A., Stiller A.M., Rihani R., Frewin C.L., Varner V.D., Gardner T.J., Cogan S.F. (2017). Thinking small: Progress on microscale neurostimulation technology. Neuromodul. Technol. Neural Interfaces.

[39] Deku F., Cohen Y., Joshi-Imre A., Kanneganti A., Gardner T.J., Cogan S.F. (2018). Amorphous silicon carbide ultramicroelectrode arrays for neural stimulation and recording. J. Neural Eng..

[40] Lewitus D.Y., Smith K.L., Landers J., Neimark A.V., Koh J. (2014). Bioactive agarose carbon-nanotube composites are capable of manipulating brain-implant interface. J. Appl. Polym. Sci..

[41] Spencer K.C., Sy J.C., Ramadi K.B., Graybiel A.M., Langer R., Cima M.J. (2017). Characterization of mechanically matched hydrogel coatings to improve the biocompatibility of neural implants. Sci. Rep..

[42] Cody P.A., Eles J.R., Lagenaur C.F., Kozai T.D.Y., Cui X.T. (2018). Unique electrophysiological and impedance signatures between encapsulation types: An analysis of biological Utah array failure and benefit of a biomimetic coating in a rat model. Biomaterials.

[43] Luan L., Wei X., Zhao Z., Siegel J.J., Potnis O., Tuppen C.A., Lin S., Kazmi S., Fowler R.A., Holloway S. (2017). Ultraflexible nanoelectronic probes form reliable, glial scar–free neural integration. Sci. Adv..

[44] Takeuchi S., Ziegler D., Yoshida Y., Mabuchi K., Suzuki T. (2005). Parylene flexible neural probes integrated with microfluidic channels. Lab Chip.

[45] Xu H., Weltman A., Scholten K., Meng E., Berger T.W., Song D. Chronic multi-region recording from the rat hippocampus in vivo with a flexible Parylene-based multi-electrode array. Proceedings of the 39th Annual International Conference of the IEEE Engineering in Medicine and Biology Society (EMBC).

[46] Mercanzini A., Cheung K., Buhl D.L., Boers M., Maillard A., Colin P., Bensadoun J.C., Bertsch A., Renaud P. (2008). Demonstration of cortical recording using novel flexible polymer neural probes. Sens. Actuators A Phys..

[47] Ludwig K.A., Uram J.D., Yang J., Martin D.C., Kipke D.R. (2006). Chronic neural recordings using silicon microelectrode arrays electrochemically deposited with a poly (3,4-ethylenedioxythiophene)(PEDOT) film. J. Neural Eng..

[48] Kozai T.D.Y., Du Z., Gugel Z.V., Smith M.A., Chase S.M., Bodily L.M., Caparosa E.M., Friedlander R.M., Cui X.T. (2015). Comprehensive chronic laminar single-unit, multi-unit, and local field potential recording performance with planar single shank electrode arrays. J. Neurosci. Methods.

